# Thiolated chitosan nanoparticles enhance anti-inflammatory effects of intranasally delivered theophylline

**DOI:** 10.1186/1465-9921-7-112

**Published:** 2006-08-24

**Authors:** Dong-Won Lee, Shawna A Shirley, Richard F Lockey, Shyam S Mohapatra

**Affiliations:** 1 Division of Allergy and Immunology and Joy McCann Culverhouse Airway Disease Center, Department of Internal Medicine, University of South Florida College of Medicine and James A. Haley Veteran's Hospital, Tampa, FL 33612, USA

## Abstract

**Background:**

Chitosan, a polymer derived from chitin, has been used for nasal drug delivery because of its biocompatibility, biodegradability and bioadhesiveness. Theophylline is a drug that reduces the inflammatory effects of allergic asthma but is difficult to administer at an appropriate dosage without causing adverse side effects. It was hypothesized that adsorption of theophylline to chitosan nanoparticles modified by the addition of thiol groups would improve theophylline absorption by the bronchial epithelium and enhance its anti-inflammatory effects.

**Objectives:**

We sought to develop an improved drug-delivery matrix for theophylline based on thiolated chitosan, and to investigate whether thiolated chitosan nanoparticles (TCNs) can enhance theophylline's capacity to alleviate allergic asthma.

**Methods:**

A mouse model of allergic asthma was used to test the effects of theophylline *in vivo*. BALB/c mice were sensitized to ovalbumin (OVA) and OVA-challenged to produce an inflammatory allergic condition. They were then treated intranasally with theophylline alone, chitosan nanoparticles alone or theophylline adsorbed to TCNs. The effects of theophylline on cellular infiltration in bronchoalveolar lavage (BAL) fluid, histopathology of lung sections, and apoptosis of lung cells were investigated to determine the effectiveness of TCNs as a drug-delivery vehicle for theophylline.

**Results:**

Theophylline alone exerts a moderate anti-inflammatory effect, as evidenced by the decrease in eosinophils in BAL fluid, the reduction of bronchial damage, inhibition of mucus hypersecretion and increased apoptosis of lung cells. The effects of theophylline were significantly enhanced when the drug was delivered by TCNs.

**Conclusion:**

Intranasal delivery of theophylline complexed with TCNs augmented the anti-inflammatory effects of the drug compared to theophylline administered alone in a mouse model of allergic asthma. The beneficial effects of theophylline in treating asthma may be enhanced through the use of this novel drug delivery system.

## Background

Asthma is a chronic inflammatory disease of the airway characterized by the infiltration of eosinophils, epithelial hyperplasia leading to hypersecretion of mucus and the presence of airway hyperresponsiveness (AHR) to a variety of stimuli [1,2]. Theophylline had been used worldwide for the treatment of asthma for several decades, but its use has recently declined owing to the increased use of inhaled glucocorticoids. Theophylline's side effects, such as nausea, headache and cardiac arrhythmias, at the dose necessary to achieve bronchodilation (plasma levels of 10 to 20 mg/L) in asthma also limit its use [1,3]. In spite of these drawbacks, theophylline remains a widely prescribed anti-asthmatic agent [4-6].

In addition, theophylline has been reported to suppress the activation of inflammatory cells, such as neutrophils and eosinophils at concentrations lower than what is required for bronchodilation [3,7,8]. Recent clinical and experimental research showed that theophylline at plasma levels of <10 mg/L still possesses anti-inflammatory and immunomodulatory properties, which may permit its use in the long-term treatment of chronic obstructive pulmonary inflammation [9,10]. The incidence of adverse side effects is minimized at this dose [1].

Chitosan, a linear polysaccharide derived from chitin obtained from crustacean shells, has emerged as a useful drug delivery matrix because of it polycationic nature, biodegradability, biocompatibility, mucoadhesiveness and ease of physical and chemical modification [11]. The interaction between cationic amino groups on chitosan and anionic moieties such as sialic and sulfonic acids on the mucus layer is responsible for its mucoadhesiveness [12]. In addition, chitosan enhances epithelial permeability through the opening of tight junctions between epithelial cells [13]. Recently, it was reported that the covalent attachment of thiol groups to polymers greatly increases their mucoadhesiveness and permeation properties without affecting biodegradability [12,14].

In this study, we hypothesized that the absorption of theophylline through bronchial mucosa could be enhanced by administration with thiolated chitosan nanoparticles (TCNs) because of their greater mucoadhesiveness and permeability properties. The anti-inflammatory effects of theophylline were measured in BALB/c mice that had been made allergic to ovalbumin. Histopathology of lung tissue after OVA challenge, eosinophilia in bronchoalveolar lavage fluid, levels of mucin production and apoptosis of lung cells were examined to evaluate the effects of theophylline.

## Materials and methods

### Materials

Chitosan (33 k*Da*) was obtained from TaeHoon Bio. Co (Korea) and used as received. The viscosity and degree of deacetylation as determined by the supplier were 2.8 cps (0.5% solution in 0.5% acetic acid at 20°C) and 90%, respectively. Thioglycolic acid, sodium tripolyphosphate, theophylline and mucin type I-S (9–17% sialic acid) and III (~1% sialic acid) were purchased from Sigma-Aldrich, USA, and used without further purification.

### Synthesis of thiolated chitosan

The chemical modification of chitosan was performed as previously described [15,16]. Chitosan (500 mg) was dissolved in 50 mL of 1.0% acetic acid. In order to facilitate reaction with thioglycolic acid (TGA), 100 mg of 1-ethyl-3-(3-dimethylaminopropyl) carbodiimide hydrochloride (EDAC) was added to the chitosan solution. After EDAC was dissolved, 30 mL of TGA was added and the pH was adjusted to 5.0 with 3 N NaOH. The reaction mixture was stirred and left for 3 h at room temperature. To eliminate the unbound TGA and to isolate the polymer conjugates, the reaction mixture was dialyzed against 5 mM HCl five times (molecular weight cut-off 10 k*Da*) over a period of 3 days in the dark, then two times against 5 mM HCl containing 1.0% NaCl to reduce ionic interactions between the cationic polymer and the anionic sulfhydryl compound.

### Preparation and characterization of chitosan nanoparticles

Chitosan suspensions of 0.2% (w/v) were prepared in 1% acetic acid. Sodium tripolyphosphate (TPP, 1.0%) was added dropwise to 6 ml of chitosan with stirring, followed by sonication with a Dismembrator (Fisher Scientific) for 10 sec at a power setting of 3 watts. The resulting chitosan particle suspension was centrifuged at 10,000 × g for 10 min. The pelleted particles were resuspended in deionized water with 10 sec sonication and lyophilized. The mean size and zeta potential of the chitosan nanoparticles were determined by photon correlation spectroscopy using a ZetaPlus particle analyzer (Brookhaven Instrument Corp., Holtsville, NY, USA).

### Adsorption of mucin by chitosan nanoparticles and mucin assay

Mucoadhesiveness was calculated as the amount of mucin adsorbed by 2 mg of chitosan nanoparticles in a certain time period. Chitosan nanoparticle suspensions (4 mg/mL) were mixed with type I-S or type III mucin solutions (0.5 and 1 mg/mL), vortexed, and incubated at 37°C for 1, 6, 12 and 18 h. After adsorption, the suspensions were centrifuged at 10,000 × g for 10 min and free mucin was measured in the supernatant by a colorimetric method using periodic acid/Schiff (PAS) staining [17]. Schiff reagent was prepared by diluting pararosaniline solution (40 g/L in 2 M HCl, Sigma) with water to give a final concentration of 1.0%. Sodium bisulfite (80 mg) was added to 5 mL of Schiff reagent and the resultant solution was incubated at 37°C until it became colorless or pale yellow. Periodic acid solution was freshly prepared by adding 10 μL of 50% periodic acid to 7 mL of 7% acetic acid. Supernatants were mixed with 100 μL of dilute periodic acid and incubated for 2 h at 37°C. Then, 100 μL of Schiff reagent was added at room temperature, and after 30 min the absorbance was measured at 560 nm. The amount of mucin adsorbed by the chitosan nanoparticles was determined by subtracting the concentration of mucin in solution after adsorption from that before. Mucin standards (0.1, 0.25 and 0.5 mg/mL) were measured by the same procedure and a standard calibration curve was prepared.

### Nasal delivery of theophylline

BALB/c mice (n = 4) were sensitized by i.p. injection of 50 μg ovalbumin (OVA, Sigma Grade V,) precipitated with alum (Imject, Pierce) on day 1. On day 8 and 14 mice were challenged intranasally with 50 μg of OVA. Theophylline (30 mM) was added to the chitosan nanoparticle suspension (4 mg/mL) and allowed to adsorb to the nanoparticles for 12 h at room temperature. Mice were given 50 μL of theophylline with or without chitosan nanoparticles intranasally on days 15, 16 and 17. Control mice were given PBS only.

### Determination of eosinophil number in bronchoalveolar lavage (BAL) fluid

Mice were euthanized on day 22 and lungs were lavaged with 500 μL of PBS introduced through the trachea. BAL fluid was centrifuged at 300 × g for 5 min, rinsed and cells resuspended with PBS. Aliquots of the cell suspension were applied to slides by Cytospin (Shandon Scientific) at 500 × g for 5 min allowed to air dry and stained with modified Wright's stain (Hema-3, Fisher Scientific). The eosinophils were determined morphologically and a minimum of 200 cells per slide were counted under the microscope.

### Lung histology, mucin production and apoptosis assay

Mice were euthanized and their lungs were perfused with PBS, removed and fixed in 4% buffered formalin. Lungs were embedded in paraffin, sectioned and stained with hematoxylin and eosin. A semi-quantitative microscopic evaluation of inflammatory cells in the entire lung section was done by persons blinded to the type of treatment. Inflammatory infiltrates were assessed morphologically for location, numbers and cell types [18,19]. Epithelial changes were scored as: 0 = no change, 1 = increased epithelial cell cytoplasm without desquamation, 2 = epithelial desquamation without bronchial exudates composed of inflammatory cells, 3 = bronchial exudates composed of desquamated epithelial cells and inflammatory cells. For peribronchovascular infiltrates: 0 = no infiltrate, 1 = infiltrate up to 4 cells thick in most vessels, 2 = infiltrate from five to seven cells thick in most vessels, 3 = infiltrate greater than 7 cells thick in most vessels. For interstitial-alveolar cell infiltrates: 0 = no infiltrate, 1 = mild generalized increase in cell mass of the alveolar septa without thickening of the septa or significant airspace consolidation, 2 = dense septal mononuclear infiltrates with thickening of septa, 3 = significant alveolar consolidation in addition to interstitial inflammation.

Goblet cells were identified using a monoclonal antibody to the marker protein mucin 5AC (MUC5AC, Lab Vision Co) visualized immunohistochemically. The presence of apoptotic cells was determined by examining sections using the TUNEL (terminal deoxynucleotidyl transferase dUTP nick end-labeling) assay. Lung sections from paraffin blocks were dewaxed in xylene, rehydrated to PBS and fixed with 4% paraformaldehyde for 15 min at room temperature. Sections were washed three times in PBS, permeablized for 15 min with 0.1% Triton X-100 and incubated with the TUNEL reagent at 37°C for 1 h. The reaction was terminated by rinsing slides once with 2× SSC and three times in PBS. The lung sections were examined under a fluorescent microscope and photographed.

### Statistical analysis

Pairs of groups were compared by Student's *t *test. Differences between groups were considered significant at *p *< 0.05. Values for all measurements are expressed as means ± SD.

## Results

### Characterization of thiolated chitosan nanoparticles

**Figure 1:**
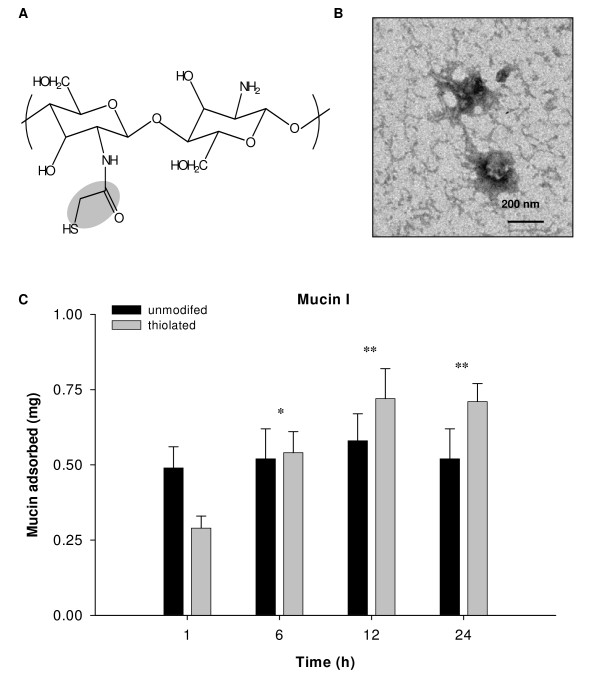
Chemical structure of chitosan-thioglycolic acid conjugate **(A) **and transmission electron micrograph of chitosan nanoparticles following ionic crosslinking **(B)**. Adsorption kinetics of mucin I-S with unmodified and thiolated chitosan nanoparticles **(C)**. The value of mucin adsorbed represents the amount of mucin per 2 mg of chitosan nanoparticles. The experiments were repeated twice and results are expressed as mean ± S.D (**P < 0.01, *P < 0.05 relative to 1 h).

Lyophilized thiolated chitosan (chitosan-thioglycolic acid conjugates, Fig. 1) is a fibrous white powder easily soluble in water. The degree of thiolation was determined by reacting the thiol groups with Ellman's reagent (5,5'-dithiobis(2-nitrobenzoic acid) and reading the absorbance at 412 nm spectrophotometrically. The content of thiol groups on thiolated chitosan was found to be 17–30 μM/g. Unmodified and thiolated chitosan nanoparticles prepared by ionic crosslinking with sodium tripolyphosphate have a diameter of 220 ± 23 nm. The zeta potential is a measure of the charge density on particles and can affect their capacity for aggregation and interaction with charged surfaces such as cells or membranes. Because of its amino groups, unmodified chitosan has a net positive charge and zeta potential of 22.7 ± 4 mV. Thiolation reduces the charge somewhat so that TCNs have a zeta potential of 15.3 ± 2 m*V*.

### Mucoadhesiveness of chitosan nanoparticles

Mucin adsorption by TCNs was measured as a way to test their ability to bind to the mucosal surfaces of the body (mucoadhesiveness). Mucin, a glycoprotein, is the major component of the mucus that coats the cells lining the surfaces of the respiratory, digestive and urogenital tracts [20]. Mucoadhesiveness was calculated as the amount of mucin adsorbed by 2 mg of chitosan nanoparticles in a certain time period. Unmodified chitosan nanoparticles adsorbed a larger amount of mucin I-S and mucin III, compared to thiolated chitosan nanoparticles after 1 h incubation (data not shown). The amount of mucin adsorbed increased with mucin concentration and percentage of sialic acid residues. Both unmodified and thiolated chitosan nanoparticles bound more mucin type I-S (containing 12% sialic acid) than mucin III (containing 1% sialic acid). Unmodified chitosan nanoparticles reached adsorption equilibrium at about 1 h at 37°C. In contrast, TCNs exhibited a more gradual increase in mucin I-S adsorption and greater binding at 12 h than unmodified chitosan (Fig. 1c).

### *In vivo *effectiveness of thiolated chitosan delivery of theophylline: treatment reduces the number of eosinophils in bronchoalveolar lavage (BAL) fluid

**Figure 2:**
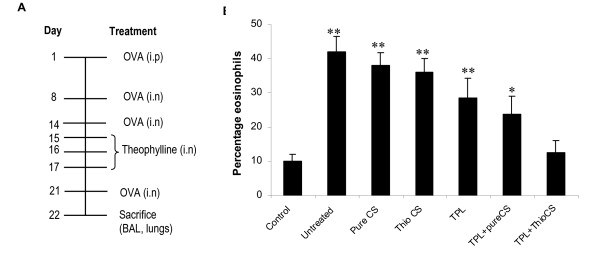
Experimental protocol used for ovalbumin-induced allergic asthma **(A) **and reduction of eosinophils in BAL fluid from theophylline (TPL)-treated mice **(B)**. BAL was performed and eosinophils were counted on day 22. The results of a representative experiment of two performed is shown. Cell counts were performed by different persons in a blinded fashion. CS denotes chitosan. (**p < 0.01, *p < 0.07 relative to TPL-thioCS).

The hypothesis of this study was that the absorption of theophylline by the bronchial epithelium and its pharmaceutical action can be enhanced by delivering theophylline as a complex with TCNs. To test this, mice allergic to ovalbumin (OVA) were challenged with OVA and then given theophylline or theophylline complexed with chitosan nanoparticles according to the protocol in Fig. 2a. Eosinophils are known to migrate to the site of an allergic reaction and to modulate the allergic inflammatory response [21]. Fig. 2b shows the effect of theophylline delivery on the percentage of eosinophils in BAL fluid. Administration of OVA increased the number of eosinophils in the airway at the subepithelial region beneath the basement membrane compared to unchallenged controls. The percentage of eosinophils in the BAL fluid was significantly reduced in the group treated with theophylline plus TCNs compared to untreated mice, or mice given unmodified chitosan, theophylline or theophylline plus unmodified chitosan.

### Thiolated chitosan nanoparticles (TCNs) enhance anti-inflammatory effects of theophylline

**Figure 3:**
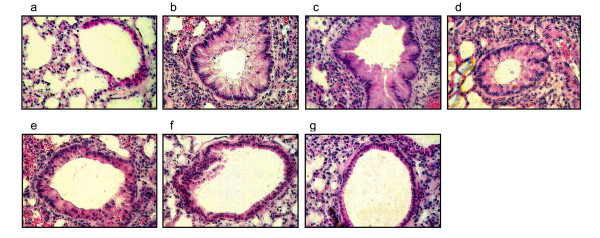
Histological analysis of mouse lung sections after theophylline treatment: (a) negative control, (b) untreated (OVA-challenged), (c) unmodified ('pure') chitosan nanoparticles, (d) Thiolated chitosan, (e) theophylline, (f) theophylline plus unmodified chitosan nanoparticles, (g) theophylline plus TCNs. On day 22, lungs were removed, sectioned and stained with hematoxylin/eosin. Examination of tissue sections was performed by different persons in a blinded fashion. The results of a representative experiment of 3 slides for each section are shown.

The effects of theophylline on OVA allergen-induced histopathology in lung sections from treated mice are shown in Fig. 3. The lung sections from OVA-challenged allergic mice clearly show epithelial damage, luminal narrowing due to airway wall edema and obstruction with excess mucus which are typical of bronchial inflammation (Fig. 3b). Administration of chitosan or thiolated chitosan nanoparticles to the sensitized and challenged mice did not produce anti-inflammatory effects, however, theophylline treatment produced a moderate reduction in lung pathology. The mice treated with theophylline plus TCNs, however, showed a considerable reduction in pulmonary inflammation, decreased epithelial damage, reduced goblet cell hyperplasia and fewer infiltrating inflammatory cells in the interstitial and peribronchovascular regions compared to the other groups (Fig. 3g).

**Figure 4:**
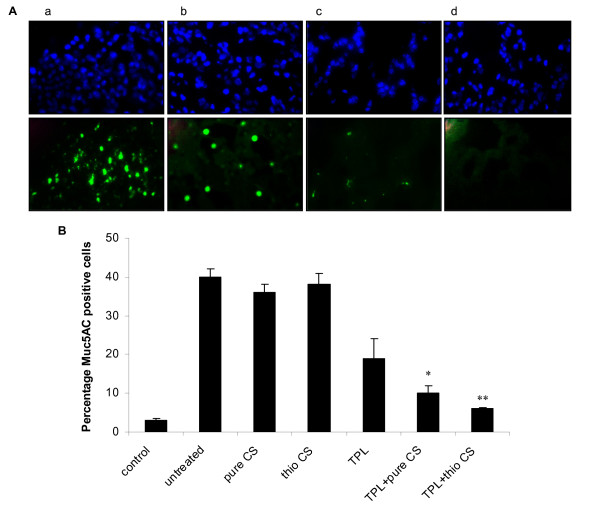
Expression of MUC5AC in unstained lung sections **(A)**: (a) untreated (OVA-challenged), (b) theophylline, (c) theophylline plus pure chitosan nanoparticles, (d) theophylline plus TCNs (top panel: nuclei stained with DAPI, bottom panel: FITC-labeled muc5AC) and semiquantitative analysis of muc5AC in unstained lung sections **(B)**. The results of a representative experiment of two performed is shown. **p < 0.01, *p < 0.05 compared with TPL group.

Unstained lung sections were examined for expression of mucin by staining with antibody against MUC5AC (Fig. 4A). MUC5AC is the most prevalent mucin subtype in mucus and is known to be specifically produced by airway goblet cells [2,22]. OVA-allergic mice challenged with OVA produce excess mucus in the airways and this hypersecretion is associated with an increased number of goblet cells. Chitosan or thiolated chitosan did not reduce mucus hypersecretion; but less immunoreactive MUC5AC was observed in mice given theophylline treatment alone (Fig. 4A panelb), indicating that the drug was able to inhibit goblet cell mucus production. The effect of theophylline on the reduction of mucus hypersecretion was further enhanced through delivery by TCNs (Fig. 4A paneld), suggesting that TCNs with enhanced mucoadhesiveness increased the absorption of theophylline and its anti-inflammatory effects (Fig. 4B).

**Figure 5:**
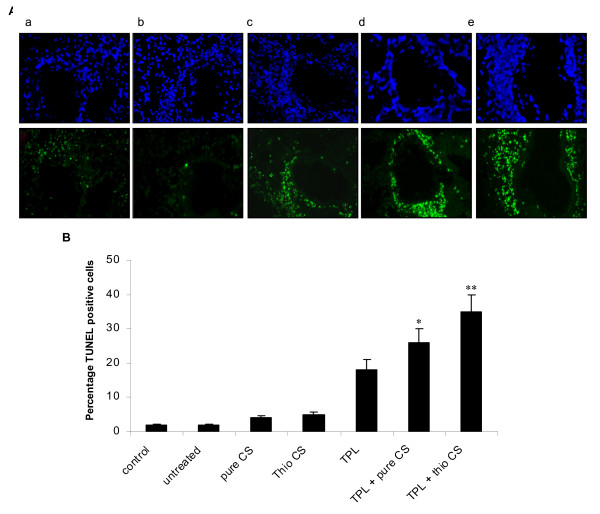
**(A) **Apoptosis in unstained lung sections by TUNEL assay: (a) untreated (OVA-challenged), (b) unmodified (pure) chitosan nanoparticles, (c) theophylline, (d) theophylline plus pure chitosan nanoparticles, (e) theophylline plus TCNs (top panel: nuclei stained with DAPI; bottom panel: apoptotic cells stained with FITC). **(B) **semiquantitative analysis of TUNEL positive cells. Examination of tissue sections was performed by different persons in a blinded fashion. The results of a representative experiment of two performed is shown. **p < 0.01, *p < 0.05 compared with TPL.

Allergic inflammation of the lung is characteristically accompanied by infiltration of immune system cells such as eosinophils which can increase the cytopathological effects. A decrease in eosinophilia could reduce the inflammatory effects of allergen challenge. To test this hypothesis, unstained lung sections were examined for apoptosis of inflammatory cells using the TUNEL assay. No apoptotic (TUNEL-positive green fluorescent) cells were observed in lungs from untreated mice or mice treated with unmodified or thiolated chitosan nanoparticles. Treatment with theophylline caused a modest increase in number of apoptotic cells in the subepithelial region beneath the airway basement membrane (Fig. 5, panel *c*). Delivery of theophylline by TCNs, however, significantly enhanced apoptosis (Fig. 5, panel *e*). These results clearly demonstrate that theophylline is capable of inducing apoptosis of lung cells and that its anti-inflammatory effects are enhanced when delivered with mucoadhesive TCNs.

## Discussion

In this study we examined the anti-inflammatory effects of the anti-asthma drug theophylline in ovalbumin-allergic mice when delivered intranasally as a complex with TCNs. Nasal drug delivery is considered to be a promising technique because of the large surface area in the nose, high total blood flow, avoidance of first-pass metabolism, quicker onset of pharmacological activity, porous endothelial basement membranes, and ready accessibility [23]. The nasal cavity presents an excellent route for chitosan-based drug delivery systems because of the attraction of the particles to mucus. Chitosan has anti-inflammatory and immunostimulatory activity, and is able to increase transcellular and paracellular transport across the mucosal epithelium [24]. Chitosan drug delivery systems employing nano/microspheres, liposomes, emulsions and gels have been demonstrated to have good bioadhesive characteristics for the nasal mucosa. Thus, it is reasonable to postulate that bioavailability, adsorption and residence time of drugs administered via the nasal route would be increased by using a drug delivery system such as chitosan nanoparticles with high mucoadhesion.

Various natural and synthetic polymers have been found to have mucoadhesive properties. Their capability to interact with glycoproteins in mucus is mainly based on noncovalent ionic interactions, van-der Waal's forces and hydrogen bonds [25]. The mucoadhesiveness of chitosan is a result of electrostatic interaction between negatively charged mucin and positively charged chitosan [15,26], and the ionization of sialic acid residues is thought to be responsible for the negative charge of mucin. This was supported by our observation that both unmodified and thiolated chitosan nanoparticles bound mucin I-S (containing 12% sialic acid) more than mucin III (containing 1% sialic acid) (data not shown).

Unmodified chitosan nanoparticles have a higher zeta potential than thiolated chitosan and therefore a greater positive charge. This may account for the greater initial adsorption of mucin by the unmodified particles at the 1 h time point (Fig. 1C). The reduced mucin adsorption on thiolated chitosan is attributed to the diminished positive charge of thiolated chitosan and subsequently decreased electrostatic interaction with negatively charged mucin. While unmodified chitosan nanoparticles reached adsorption equilibrium at 1 h of incubation with mucin, TCNs exhibited a steady increase in mucin adsorption up to 12 hours. This enhanced mucoadhesiveness may be attributed to the formation of disulfide bonds between thiol groups on chitosan and cysteine-rich subdomains of mucus glycoproteins [16,27]. Thus, TCNs are an excellent candidate drug delivery platform for the mucus-rich bronchial epithelium [2,19,28]. Based on previous studies showing that hydrogen bonds formed between the amino groups of chitosan and the carbonyl groups of theophylline, it was reasoned that theophylline first adsorbs onto the chitosan nanoparticle surface then diffuses into the interior of the particles during the incubation of theophylline in the suspension of chitosan nanoparticles, [29], where interaction with bioadhesive particles occurs in the wet state [30].

Eosinophils are an important effector cell in the inflammatory response to allergens. Increased numbers of eosinophils are detectable in bronchial biopsy specimens and in induced sputum from asthma patients [31]. Eosinophils are known to produce and release cytotoxic proteins and reactive oxygen species such as superoxide which may contribute to epithelial damage and increased bronchial hyperresponsiveness in asthmatic patients [4]. Recently, it has been recognized that theophylline can inhibit inflammatory cells and have anti-inflammatory effects on the airways of asthmatic patients [3,7,8,32]. Caramori and colleagues reported that eosinophil degranulation and release of eosinophil basic proteins were inhibited by high doses of theophylline [3]. In our work, histological examination revealed that nasal delivery of theophylline inhibited the infiltration of inflammatory cells beneath the epithelium and reduced epithelial damage in OVA-allergic mice. The reduction in eosinophils in BAL fluid was greater when theophylline was delivered by TCNs than by unmodified chitosan nanoparticles. These results clearly demonstrate that thiolation enhances the capacity of chitosan nanoparticles to effectively deliver theophylline to bronchial epithelium. It is likely that the enhanced anti-inflammatory effects of theophylline delivered by TCNs result from their increased mucoadhesiveness.

It has been suggested that apoptosis or programmed cell death of inflammatory cells may be an effective therapeutic strategy for allergic asthma. Ohta et al. reported that theophylline induced apoptosis in eosinophils and lymphocytes by means of elevation of intracellular cAMP [32]. Our results here showed that theophylline delivered by TCNs markedly induced apoptosis of inflammatory cells, presumably eosinophils and lymphocytes. It is also possible that theophylline reduced the number of eosinophils in BAL fluid in part by inhibiting their migration, although induced apoptosis of activated eosinophils at the subepithelial region beneath the basement membrane of the airway appears to be its primary activity. Induction of apoptosis of activated inflammatory cells by theophylline may be a beneficial strategy for treating allergic inflammation.

Mucus has an important role in host defense of upper and lower respiratory tracts, but represents an important cause of airway obstruction in asthma when secreted in excess [33]. Mucus hypersecretion can be an important cause of morbidity and mortality in patients with respiratory tract diseases such as asthma [34]. Muc5AC is the most common mucin secreted by goblet cells and it is the major gel-forming mucin in airway secretions. Lung sections stained with antibody against Muc5AC showed that mucus hypersecretion was remarkably inhibited by theophylline when delivered with TCNs, further supporting the observation that TCNs enhance the anti-inflammatory effect of theophylline. The mechanism underlying the increased drug delivery efficacy of TCNs is unclear. However, the most probable explanation is that the thiol groups provide chitosan with enhanced mucoadhesiveness and cell permeability via the paracellular and transcellular routes as suggested previously [30].

In summary, theophylline delivered intranasally as a complex with thiolated chitosan affords protection against the allergic lung inflammation induced by OVA sensitization and challenge in mice. The anti-inflammatory effects of theophylline were markedly enhanced when the drug was delivered by TCNs compared to unmodified chitosan or theophylline alone. The increased mucoadhesiveness of TCNs provides an effective mucosal drug delivery platform and a novel strategy to enhance the beneficial actions of theophylline in treating allergic inflammation.

## Authors' contributions

DL designed the study, performed all experiments and drafted the manuscript. SS participated in the interpretation of data and drafting the manuscript. RL and SM conceived the study and participated in its design and helped to draft the manuscript. All authors read and approved the final manuscript.

**Table 1:** A semiquantitative analysis of lung damage by histopathological scores.

	Negative control	Untreated	Pure CS	Thio-CS	TPL	TPL + Pure CS	TPL + Thio-CS
Epithelium	0.8 ± 0.3	2.5 ± 0.3	2.1 ± 0.2	2.0 ± 0.2	1.8* ± 0.2	1.6* ± 0.2	1.2** ± 0.2
Peribroncho-vascular infiltrate	0.4 + 0.2	2.1 ± 0.4	2.0 ± 0.4	1.9 ± 0.1	1.4* ± 0.2	1.4* ± 0.4	0.8** ± 0.2
Interstitial-alveolar cell infiltrate	0.7 ± 0.2	2.3 ± 0.3	2.0 ± 0.4	2.0 ± 0.2	1.6 ± 0.3	1.4* ± 0.2	0.9** ± 0.2

**p < 0.01 compared with the untreated group

*p < 0.05 compared with the untreated group
